# Differential Compliance of Patient-Reported Outcomes in a Multicenter Hand and Wrist Surgery Registry in Switzerland

**DOI:** 10.1097/PRS.0000000000012682

**Published:** 2025-12-16

**Authors:** Maurizio Calcagni, Myrna Gunning, Olga Politikou, Inga S. Besmens

**Affiliations:** Zurich, Switzerland; From the 1 Hand Institute Zurich; 2 Department of Plastic Surgery and Hand Surgery, University Hospital Zurich.

## Abstract

**Background:**

Outcome registries are essential for assessing treatment effectiveness in hand and wrist surgery, in which both patient-reported outcome measures (PROMs) and clinician-reported outcome measures are commonly used. Consistent patient participation is essential for generating valid and generalizable data. This study investigates age- and sex-related differences in PROM compliance within a multicenter Swiss registry.

**Methods:**

Registry data from patients treated between 2020 and 2024 were analyzed. Patients were stratified by age (18 to 30 years, 31 to 40 years, 41 to 50 years, 51 to 60 years, 61 to 70 years, and 70 years or older) and sex. Compliance with PROM completion was evaluated at baseline and 3 and 12 months after treatment.

**Results:**

PROM compliance declined over time, with the most pronounced decrease observed in the 18 to 30 years age group (over 90% loss at 12 months). The 61 to 70 years age group showed the highest sustained compliance. Across all age categories, women demonstrated consistently higher completion rates than men.

**Conclusions:**

Compliance with PROMs varies significantly by age and sex, leading to potential data bias in outcome registries. Younger patients and men are underrepresented at follow-up, which may compromise the validity and generalizability of registry-based findings. Targeted interventions are needed to enhance participation in these groups and improve the quality of clinical outcome data.

Outcomes registries play a vital role in evaluating the effectiveness of medical treatments and guiding clinical decision-making.^1^ In the field of hand and wrist surgery, capturing both patient-reported outcome measures (PROMs) and clinician-reported outcome measures (CROMs) provides a comprehensive view of treatment success.^2^

PROMs have become essential tools for capturing the patient’s perspective in clinical care and research, ensuring patient-centered evaluation of treatment effectiveness.^3^ In hand surgery, PROMs such as the Disabilities of the Arm, Shoulder, and Hand questionnaire (DASH), QuickDASH, and Michigan Hand Outcomes Questionnaire are widely used.^4^ The growing international development and validation of hand-specific PROMs, such as the HAND-Q, reflects the increasing emphasis on systematically incorporating patient-reported outcomes into both research and clinical registries to improve quality of care and enable meaningful international comparisons.

Sustained patient and physician engagement in such registries is essential for collecting reliable and longitudinal data.^5^ Without consistent participation, the quality of the data diminishes, potentially leading to biased interpretations of treatment efficacy. Understanding the factors that influence compliance is therefore key to enhancing the accuracy and applicability of registry-based research.^6^ Differential compliance in completing these measures can skew results, limiting the generalizability and utility of the registry’s findings. Previous studies have suggested that compliance with follow-up questionnaires tends to decline over time, with variations observed across different demographic groups.^7^

In this study, we examine variations in compliance within a structured hand and wrist surgery outcomes registry in Switzerland, focusing on differences by age and sex.

## PATIENTS AND METHODS

### Study Design and Setting

This study was conducted using data from a multicenter hand and wrist surgery outcomes registry in Switzerland, established in 2020. The registry was designed in accordance with the International Consortium for Health Outcomes Measurement (ICHOM) guidelines for hand and wrist surgery, ensuring standardized data collection and reporting across multiple sites. This study was approved by the Cantonal Ethics Committee of Zurich, Switzerland (approval no. 2024-02013). All participants provided written informed consent in accordance with the Declaration of Helsinki. Raw data for this study are not publicly available, to preserve individual privacy under Swiss data protection laws.

### Participants

Patients who underwent hand and wrist surgery at participating centers between 2020 and 2024 were included in the registry. All patients gave written informed consent to being included in this registry. Patients were stratified into 6 age groups: 18 to 30 years, 31 to 40 years, 41 to 50 years, 51 to 60 years, 61 to 70 years, and 70 years or older. Data on sex were also collected, allowing for analysis by male and female subgroups. Sex was recorded as male, female, or nonbinary by the treating physician; however, no patient in this cohort was recorded as nonbinary. For a certain number of patients (Table 1), no sex was recorded.

**Table 1.** Compliant Patients Grouped According to Sex and Age

Sex and Age	No. of Patients	Compliant at Intake, *n* (%)	Compliant at 3 mo, *n* (%)	Compliant at 12 mo, *n* (%)
Female				
18–30 yrs	56	26 (46.4)	13 (23.2)	10 (17.9)
31–40 yrs	75	30 (40.0)	23 (30.7)	7 (9.3)
41–50 yrs	71	40 (56.3)	23 (32.4)	10 (14.1)
51–60 yrs	88	57 (64.8)	32 (36.4)	28 (31.8)
61–70 yrs	91	55 (60.4)	41 (45.1)	29 (31.9)
70+ yrs	64	41 (64.1)	22 (34.4)	12 (18.8)
Male				
18–30 yrs	228	85 (37.3)	40 (17.5)	17 (7.5)
31–40 yrs	190	84 (44.2)	37 (19.5)	23 (12.1)
41–50 yrs	142	64 (45.1)	39 (27.5)	22 (15.5)
51–60 yrs	153	81 (52.9)	42 (27.5)	29 (19.0)
61–70 yrs	92	57 (62.0)	43 (46.7)	20 (21.7)
70+ yrs	51	25 (49.0)	15 (29.4)	8 (15.7)
NA				
18–30 yrs	7	2 (28.6)	1 (14.3)	
31–40 yrs	8	5 (62.5)	2 (25.0)	0
41–50 yrs	10	4 (40.0)	4 (40.0)	0
51–60 yrs	1		1	0
61–70 yrs		1	1	0
70+ yrs		3	3	0

### Patient-Reported Outcome Measures

PROMs included standardized questionnaires assessing pain, function, and overall satisfaction with treatment according to the ICHOM Hand & Wrist Standard Set.^2^ Across tracks, the core PROMs comprised the Patient-Rated Wrist/Hand Evaluation for pain and function, health-related quality of life using the EQ-5D-5L, pain intensity using a numeric rating scale/visual analog scale, and 2 brief ICHOM items for return-to-work status and satisfaction with treatment results. For track-specific symptoms, cold intolerance was captured using the Cold Intolerance Symptom Severity questionnaire in the nerve and severe trauma contexts as specified by the Standard Set.

Compliance with PROMs was tracked at 3 key time points: baseline (pretreatment), 3 months after treatment, and 12 months after treatment.

### Data Collection and Analysis

Data were collected electronically through the registry’s online platform, which sent automated reminders to patients at the specified follow-up intervals.

Statistical analyses were performed using R software (version 3.4.3). Statistical analyses were conducted to compare compliance rates across different age groups and between sexes. Compliance rates were calculated as the percentage of participants who completed the required outcome measures at each time point. Multivariable logistic regression was performed to identify independent predictors of 12-month compliance. Variables included in the model were age group (18 to 30 years as reference), sex (male as reference), and baseline compliance status. Results are reported as odds ratios (ORs) with 95% confidence intervals (CIs).

### Statistical Power and Sample Size Considerations

Post hoc power analysis indicated that our sample size of 1335 patients provided excellent statistical power (greater than 95%) to detect the observed differences in compliance rates between sex groups (21.6% versus 13.9%) and age groups (eg, 9.3% for 18 to 30 years versus 26.8% for 61 to 70 years). The study had adequate power to detect clinically meaningful differences of approximately 1 to 2 percentage points between groups. For the multivariable logistic regression analysis, the number of events (215 patients with 12-month compliance) exceeded the recommended minimum of 10 events per predictor variable, ensuring stable model estimates.

## RESULTS

Between 2020 and 2024, a total of 1335 patients were included in the registry: 856 male, 445 female, and 34 who did not choose a binary gender (Table 1).

### Compliance

#### Overall Compliance Trends

Compliance rates for PROMs showed a general decline from baseline to the 12-month follow-up. At baseline, the overall compliance rate was 47.9% (95% CI, 45.2 to 50.6%), which declined to 28.3% (95% CI, 25.9 to 30.8%) at 3 months, and further to 16.5% (95% CI, 14.6 to 18.6%) at 12 months. Although the decreases at baseline and 3 months were not statistically significant, the progressive attrition is evident. Of note, compliance at baseline was lowest among younger male patients compared with other demographic groups, although these differences did not reach statistical significance.

Compliance varied significantly by age and sex. Specific patient compliance across age and sex is summarized in Table 2.

**Table 2.** Compliance Rates by Age Group

Age Group	Compliant at Baseline, %	Compliant at 3 mo, %	Compliant at 12 mo, %	OR (95% CI)	*P* versus Age 18 to 30 yrs
18–30 yrs	39	19	9	Reference	—
31–40 yrs	44	23	11	1.17 (0.68–2.04)	0.570
41–50 yrs	49	30	14	1.59 (0.92–2.78)	0.096
51–60 yrs	57	31	24	2.77 (1.69–4.61)	<0.001
61–70 yrs	60	46	26	3.10 (1.85–5.29)	<0.001
70+ yrs	58	34	17	1.74 (0.91–3.28)	0.087

Twelve-month compliance rates varied significantly by age group, as follows: 18 to 30 years, 9.3% (95% CI, 6.5% to 13.2%); 31 to 40 years, 11.0% (95% CI, 7.8% to 15.3%); 41 to 50 years, 14.3% (95% CI, 10.4% to 19.6%); 51 to 60 years, 23.6% (95% CI, 18.6% to 29.3%); 61 to 70 years, 26.8% (95% CI, 20.9% to 33.6%); and 70 years or older, 17.4% (95% CI, 11.5% to 25.3%). Sex-based analysis showed that women had significantly higher compliance rates (21.6% [95% CI, 18.0% to 25.6%]) compared with men (13.9% [95% CI, 11.7% to 16.4%]).

#### Compliance by Age

**Fig. 1.**
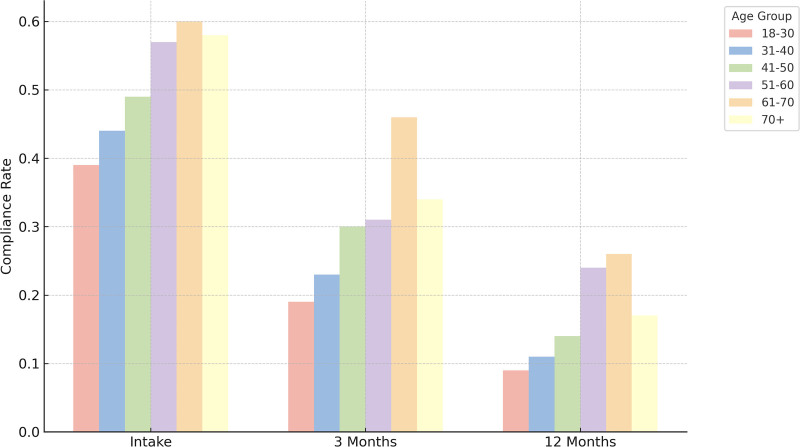
Compliance rates by age category at the 3 assessed time points.

Compliance rates decreased across all age groups from baseline to 12 months. At baseline, compliance was 39% in the 18 to 30 years group, 44% in the 31 to 40 years group, 49% in the 41 to 50 years group, 57% in the 51 to 60 years group, 60% in the 61 to 70 years group, and 58% in the 70 years or older group. By 12 months, compliance had declined to 9%, 11%, 14%, 24%, 26%, and 17%, respectively (Table 2 and Fig. 1). The most notable decline in compliance was observed in the youngest age group (18 to 30 years), where compliance decreased from 39% at baseline to 9% at 12 months—an absolute reduction of 30 percentage points, corresponding to a 77% proportional decline in compliance and over 90% attrition of participants. In contrast, the 61- to 70-year age group maintained the highest compliance rates, decreasing from 60% at baseline to 26% at 12 months (a 34 percentage point or 57% relative decline).

#### Compliance by Sex

**Fig. 2.**
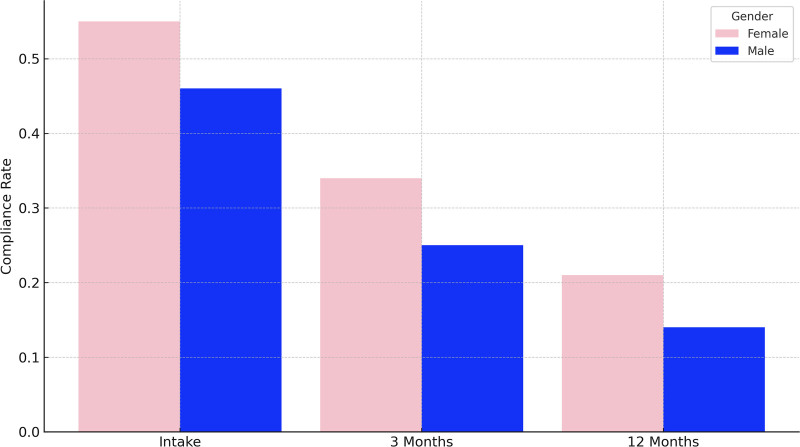
Compliance rates by sex at the 3 assessed time points.

Female participants consistently outperformed male participants in maintaining higher compliance rates at all time points (Table 3 and Fig. 2). At baseline, compliance was 55% for women compared with 46% for men. At 3 months, compliance rates were 34% for women versus 25% for men, and at 12 months, 21% for women compared with 14% for men. Although the initial sex gap in compliance narrowed slightly by the 12-month mark, women still demonstrated greater persistence in completing the required measures.

**Table 3.** Compliance Rates by Sex

Sex	Baseline, %	3 Months, %	12 Months, %	OR (95% CI)	*P* versus Male
Female	55	34	21	1.47 (1.08–2.00)	0.015
Male	46	25	14		Reference

#### Predictors of Compliance

**Fig. 3.**
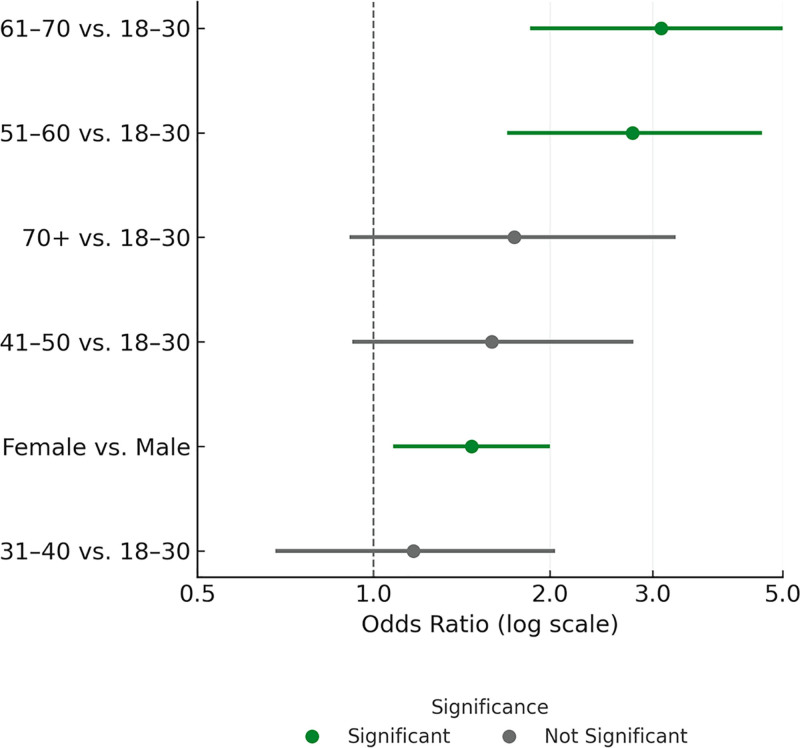
Odds of compliance at 12 months by age group and sex. Values are derived from a logistic regression model, with significant predictors indicated by confidence intervals that do not cross 1.0.

In multivariable logistic regression, age and sex were associated with compliance at 12 months (Table 4 and Fig. 3). Participants with age 51 to 60 years (OR, 2.77 [95% CI, 1.69 to 4.61]; *P* < 0.001) or 61 to 70 years (OR, 3.10 [95% CI, 1.85 to 5.29]; *P* < 0.001) had significantly higher odds of compliance compared with those with age between 18 and 30 years. Women also showed significantly higher odds of compliance (OR, 1.47 [95% CI, 1.08 to 2.00]; *P* = 0.015).

**Table 4.** Logistic Regression Predicting Compliance at 12 Months^a^

Predictor	OR (95% CI)	*P*
Age group		
31–40 vs 18–30 yrs	1.17 (0.68–2.04)	0.570
41–50 vs 18–30 yrs	1.59 (0.92–2.78)	0.096
51–60 vs 18–30 yrs	2.77 (1.69–4.61)	<0.001
61–70 vs 18–30 yrs	3.10 (1.85–5.29)	<0.001
70+ vs 18–30 yrs	1.74 (0.91–3.28)	0.087
Sex		
Female vs male	1.47 (1.08–2.00)	0.015
		

a Reference groups: age 18 to 30 years; male sex.

## DISCUSSION

The findings of this study highlight significant variations in compliance with outcome measures based on age and sex. This has wide-ranging implications for both clinical practice and the management of outcomes registries. Variations in compliance mean that certain demographic groups (eg, older patients or women) may be overrepresented in the outcomes data. This imbalance can introduce bias into the registry data, potentially leading to skewed interpretations of treatment effectiveness, patient satisfaction, and long-term outcomes. Also, longitudinal studies rely on consistent follow-up to track patient outcomes over time. Significant variations in compliance can result in incomplete data, making it difficult to perform robust longitudinal analyses. This can hinder efforts to identify trends, such as the durability of treatment effects or the emergence of late complications, across different age and sex groups. Therefore, understanding variations in patient adherence to registries is essential for ensuring that the data collected are comprehensive, accurate, and truly reflective of the diverse patient population undergoing hand and wrist surgery.

PROMs showed a general decline from baseline to the 12-month follow-up, which may reflect a lack of continuous engagement strategies to keep patients motivated and informed about the importance of their ongoing participation. The World Health Organization has highlighted poor adherence as a significant issue requiring improvement strategies.^8^ Without regular reminders or reinforcement of the value of their data for improving treatment outcomes, patients may lose interest in participating in the registry over time. Providing regular updates on the impact of their contributions and how the data are used to enhance care can reinforce their commitment. Personalized communication, such as follow-up calls or tailored messages, can also help maintain engagement.

The most notable decline in compliance was observed in the youngest age group. Younger adults often have multiple competing priorities, such as education, career development, and social activities, which may lead to lower prioritization of their health, especially after the immediate postoperative period.^9^ In addition, although this age group is typically more accustomed to digital communication, they may also experience digital fatigue due to the constant influx of notifications and digital tasks. Traditional follow-up methods, such as emails or online surveys, might be easily overlooked or deprioritized among other digital interactions.^10^ Although Panahi et al.^11^ showed that younger patients are generally compliant with health care provider recommendations, they may perceive themselves as being in better overall health and more resilient, leading to a lower perceived need for continued monitoring and follow-up, especially if they feel fully recovered.

Baseline compliance was already lowest among younger men. Although these baseline differences were not statistically significant, they may reflect demographic patterns in health survey participation. Previous studies have shown that young adult men are generally less likely to engage in preventive health behaviors and less inclined to complete health questionnaires compared with women or older individuals.^12^ This reduced baseline engagement may predispose younger men to further attrition during follow-up and underscores the need for tailored strategies that address initial participation as well as sustained compliance.

In contrast, the oldest age group (61 to 70 years) maintained the highest compliance rates. Individuals in this group often have a heightened awareness of their health and are more proactive in managing it.^13^ They are generally more attuned to the potential risks and complications associated with aging and therefore place a higher priority on following medical advice and engaging in follow-up care. Compared with younger individuals who may be juggling work, family, and other responsibilities, those in the 61- to 70-year age group often have more time to devote to health-related activities, including completing online surveys.^14^ This reduced time pressure could contribute to their higher compliance rates. In addition, this age group may have a higher level of trust in medical professionals and the health care system, making them more likely to follow through with requests from their health care providers.^15^

Analysis by sex revealed that women consistently outperformed men. Research has consistently shown that women are more proactive in managing their health compared with men.^12^ Women are generally more likely to seek medical care, adhere to treatment plans, and participate in health-related activities, including completing follow-up surveys.^16^ This greater engagement in health management is likely reflected in the higher compliance rates observed in this study. Social and cultural factors, such as caregiving roles, may also influence women’s higher compliance rates. Women often assume caregiving responsibilities, which can reinforce habits of monitoring health outcomes and adhering to medical advice, including completing follow-up surveys.^17^

This study has several limitations. First, the analysis was limited to participants who were already engaged in the registry, which may introduce selection bias. In addition, the reasons for noncompliance were not systematically recorded, making it difficult to identify specific barriers to participation. The study also focused on a single geographic region, which may limit the generalizability of the findings to other populations.

## CONCLUSIONS

This study demonstrates a significant decline in compliance with PROMs over time in a multicenter hand and wrist surgery registry, with younger age groups and men being particularly prone to noncompliance. These findings underscore the need for tailored strategies to improve long-term engagement in outcomes registries, particularly among younger and male patients. Future efforts should focus on enhancing patient engagement through targeted interventions, such as personalized follow-up strategies and improved communication methods, to ensure comprehensive and reliable data collection in hand and wrist surgery outcomes. In addition, acknowledging these variations allows for more nuanced data interpretation. Researchers and clinicians can adjust their analyses to account for the demographic characteristics of respondents, ensuring that the findings are interpreted in the appropriate context.

## DISCLOSURE

The authors declare no conflicts of interest related to the content of this article. No financial support or funding was received for this work.

## DATA AVAILABILITY

Raw data for this study are not publicly available, to preserve individual privacy under Swiss data protection laws.
